# Orthostatic Hypertension in Children: An Update

**DOI:** 10.3389/fped.2020.00425

**Published:** 2020-07-29

**Authors:** Yang Hu, Hongfang Jin, Junbao Du

**Affiliations:** ^1^Department of Pediatrics, Peking University First Hospital, Beijing, China; ^2^Research Unit of Clinical Diagnosis and Treatment of Pediatric Syncope and Cardiovascular Diseases, Chinese Academy of Medical Sciences, Beijing, China; ^3^Key Laboratory of Cardiovascular Sciences, Ministry of Education, Beijing, China

**Keywords:** orthostatic hypertension, orthostatic intolerance, pediatrics, pathogenesis, diagnosis, treatment

## Abstract

The concept of orthostatic hypertension in children was first proposed in 2012. The pathogenesis is not clear by now. Orthostatic hypertension is one of the important causes of orthostatic intolerance in children and is related to the development of essential hypertension in the future. It is commonly seen in older children, with dizziness and syncope as their main clinical manifestations. Non-drug therapy is the commonly used treatment strategy, which is effective to improve the orthostatic intolerance symptoms. In this paper, we reviewed the clinical studies on the pathogenesis, clinical characteristics, diagnostic criteria, and treatment of orthostatic hypertension in children, aiming to provide new insights for the future studies on pediatric orthostatic hypertension.

## Introduction

Orthostatic hypertension (OHT) refers to a significant increase in blood pressure (BP) when in the upright position compared to the supine position, and it reflects the abnormal regulation of BP during postural changes. In adults, OHT is often seen in patients with essential hypertension or type 2 diabetes, and it is considered a risk factor for cardiovascular and cerebrovascular diseases and is related to target organ damage and poor prognosis (1–3). Pediatric OHT was first reported in 2012 by Zhao et al., and they described that most children with OHT were in puberty and presented dizziness and syncope as their main clinical manifestations, which were often induced by sudden postural changes or prolonged standing (4). At present, OHT is recognized as an important cause of orthostatic intolerance (OI) in children. Studies also showed that OHT could increase the risk of hypertension in young adults (5). In this paper, the pathogenesis, clinical features, diagnostic criteria, and treatment of OHT in children were reviewed.

## Pathogenesis

To date, the pathogenesis of OHT has been unclear. Recently published studies reported that sympathetic overactivation in the upright posture was involved in the pathogenesis of OHT in children; this has been demonstrated by a significant decrease in sequential baroreflex sensitivity (BRS) and a remarkable increase in the low frequency/high frequency (LF/HF) ratio of R-R interval variability when switching from supine position to upright position in children with OHT compared with healthy controls (6). Sequential BRS which is the correlation between the R-R interval and beat-to-beat systolic BP (SBP) mainly reflects vagal activity in the baroreflex, while the increased LF/HF ratio of R-R interval variability reflects sympathetic predominance (6). Therefore, the changes in BRS and the LF/HF ratio when shifting from the supine to standing position in children with OHT indicated more significant vagal withdrawal and sympathetic activation in the upright posture. In addition, Zhao et al. found that children with postural tachycardia syndrome (POTS) and OHT showed higher plasma antidiuretic hormone (ADH) levels compared with children with POTS alone, and the Pearson correlation analysis showed that the plasma ADH level was positively correlated with upright SBP; thus, the increase in ADH might also be involved in the development of OHT in children (7).

Furthermore, a previous study reported that plasma nitric oxide (NO) levels and nitric oxide synthetase (NOS) activities of children with OHT were significantly lower than those of the control group, and plasma NO levels were negatively correlated with the increases in SBP from supine to upright position. This phenomenon suggests that vascular endothelial injury reflected by NO decrease might participate in the pathogenesis of OHT in children (8). However, whether vasodilation dysfunction occurs in children with OHT is unclear. In addition, Sun et al. discovered that serum 25-hydroxyvitamin D levels in children with OHT were significantly lower than those in healthy controls. Since vitamin D could affect the cardiovascular system through a variety of mechanisms, such as inhibiting the renin-angiotensin-aldosterone system (RAAS) and affecting the balance between sympathetic tone and vagal activity, they speculated that vitamin D deficiency might be associated with pediatric OHT (9).

In studies on adults, the mechanisms for OHT included elevated norepinephrine levels in plasma, abnormal baroreflex, and changes in vascular properties (10–14). A recent study also showed that standing posture was associated with the elevation of BP after upright (15). However, the differences in the mechanisms of pediatric OHT and adult OHT merit further exploration.

## Clinical Significance

OHT is one of the important causes of OI in children. Kang et al. conducted a head-up tilt test (HUTT) among 2089 children with OI symptoms, such as syncope, headache, dizziness, and chest tightness; they found that 23.84% of all subjects showed a positive response to OHT (16). Recurrent symptoms of OI greatly impact quality of life and academic performance in children and adolescents (4). Anxiety about OI symptoms, especially syncope episodes, might lead to psychological burdens among children as well.

Moreover, pediatric OHT potentially increases the risk of suffering from adulthood hypertension. The Coronary Artery Risk Development in Young Adults (CARDIA) study showed a significant increase in the risk of developing hypertension in young adults with OHT compared with those without OHT (12.4 vs. 6.8%, *P* < 0.01) (5). Frequent fluctuations in BP might lead to damage in vascular endothelial function, causing the remodeling of small vessels, and hypertension (17). In addition, OHT has been shown to be associated with masked hypertension (18, 19). Therefore, children diagnosed with OHT need long-term follow-up.

## Risk Factors

Overweight and obesity are currently recognized as risk factors for essential hypertension in children and adolescents (20), but there are not enough robust studies to clarify the relationship between obesity and pediatric OHT. Recently, by comparing the height, weight, and BMI of 76 children with OHT and 76 healthy children, Zhang et al. concluded that the BMI was higher in the OHT group than in the control group only in 13-year-old boys, while there was no significant difference in BMI between the groups among 7–12 years old children (21). As the sample size was relatively small, their results need to be further tested by larger investigations. A previous study showed that insufficient water intake and decreased sleep duration were risk factors for POTS in children (22), and lack of sleep also increased the risk of developing essential hypertension in children and adolescents (23); however, it is unknown whether water intake and sleep duration are associated with pediatric OHT.

## Clinical Chracteristics

OHT mainly occurs in older children. In the study from Zhao et al., most of the children with OHT were in puberty (the average age was 11.8 ± 2.7 years old) (4). Another study reported that OHT was mainly found in boys older than 12 years old (16). Unlike adults, children with OHT might suffer from obvious and diverse OI symptoms, such as dizziness, headache, chest tightness, chest pain, palpitation, nausea, and even syncope, which are often induced by predisposing factors, such as sudden postural changes, prolonged standing, or sitting, emotional stress and a stuffy environment (4).

Comorbidity with POTS is not uncommon in patients with OHT. Robertson reported that over 20% of patients with POTS also had OHT (24). Zhang et al. studied the clinical characteristics of children with POTS and OHT and discovered that their supine SBP was lower than that of children with POTS alone, and headache was more commonly reported in the POTS + OHT group (25). Zou et al. reported that some children with OHT also showed a hemodynamic response of vasovagal syncope (VVS) in the HUTT (26). Therefore, OHT, POTS, and VVS, which are three important causes of OI in children, may partly overlap in pathogenesis and clinical manifestations.

Regarding the circadian rhythm of BP in children with OHT, Liu et al. monitored the 24-h ambulatory BP of 43 children diagnosed with OHT and detected that 72.5% of the children with OHT showed a “non-dipper” phenotype, which was speculated to be related to the increase in sympathetic nervous activity (27). However, in studies of adults or elderly patients with OHT, the dominant BP type was shown to be the “extreme dipper,” which was characterized by a greater than normal decrease in SBP at night. In one study, Kario et al. reported that in elderly patients with essential hypertension, 72% of the extreme dippers had OHT, significantly higher than dippers and non-dippers (11 and 9%, respectively) (28). Therefore, there might be differences in the pathogenesis of pediatric OHT and adult OHT.

## Diagnosis

The methods for diagnosing OHT mainly include the active standing test, the HUTT, BP self-measurement at home and 24-h ambulatory BP monitoring. OHT can be diagnosed based on BP elevation from supine to upright or the absolute value of upright BP (1). The active standing test is easy and safe to operate, and the HUTT is often used for differential diagnosis of the causes of OI in children. Since the HUTT has the risk of inducing syncope, written informed consent should be obtained from the parents or guardians in advance, and appropriate protective measures should be taken during the test. Although the bias of “white-coat hypertension” can be effectively eliminated by home BP self-measurement or 24-h ambulatory BP monitoring, there are few reports of using these methods for the diagnosis of pediatric OHT due to the difficulties in practice.

In studies of adult patients, the majority of researchers defined OHT as an increase in SBP of at least 20 mmHg or an increase of diastolic BP (DBP) of at least 10 mmHg from supine to standing (1). For children and adolescents, a large cross-sectional investigation identified the 95th percentile of BP increase from supine to upright and the 95th percentile of upright BP values in children aged 6–18 years old (29). Based on this research, the diagnostic criteria for pediatric OHT were suggested as follows: normal supine BP; and increased SBP ≥ 20 mmHg and/or increased DBP ≥ 25 mmHg (in children 6–12 years old) or increased DBP ≥ 20 mmHg (in adolescents 13−18 years old), or BP ≥ 130/90 mmHg (in children 6−12 years old) or ≥ 140/90 mmHg (in adolescents 13–18 years old) during the initial 3 min of the standing test or the HUTT (30, 31).

## Treatment

Only non-drug treatments for pediatric OHT have been reported in the literature until now, including health education, autonomic nervous function exercise, and wearing tights. Health education includes teaching children to increase water intake and to avoid sudden postural changes or prolonged standing; psychological assistance is also provided. Liu et al. reported that health education had an effect on children with OHT, with a significant decrease in upright DBP (32). Autonomic nervous function exercise, including standing training, skin autonomic nervous training, abdominal breath training, and aerobic exercise, can help in reducing episodes of OI symptoms (33). In addition, sympathetic activation induced by decreased cardiac output after standing was thought to be one of the pathogenic mechanisms of OHT in adults, and wearing tights might increase the returned blood volume and cardiac output in the upright position (34). A study by Streeten et al. showed that the upright DBP of OHT patients significantly decreased after wearing an inflated pressure suit over the pelvis and lower limbs (34).

Wang et al. performed conventional treatment (including health education, standing training, and skin autonomic nervous exercise) on 61 children with OHT and found that the cumulative symptom rates at 1, 3 months, 1, 3, and 5 years were 57.1, 42.9, 33.3, 11.1, and 2.2%, respectively, indicating that conventional treatment was effective in improving the OI symptoms of children with OHT (35). However, follow-up data on supine and upright BPs were lacking.

Some studies have examined drug therapy in elderly patients with OHT. Oral administration of alpha-adrenergic blockers could significantly reduce orthostatic BP increase in OHT patients without affecting basic BP (10). In another clinical study, taking doxazosin at bedtime markedly reduced orthostatic BP in the OHT group, and the reduction in orthostatic BP was significantly correlated with the decrease in the urinary albumin/creatinine ratio, which reflected that the administration of doxazosin might consequently prevent target organ damage (36). However, there are no drug treatments recommended for pediatric OHT to date.

In conclusion, childhood OHT is a condition of concern that deserves attention. It mainly occurs in older children and is characterized by significantly increased SBP and/or DBP after standing. The pathogenesis and risk factors for pediatric OHT need to be further explored to better prevent and treat the disease. Long-term follow-up is required to determine the natural history of OHT in children, as well as the percentage who may develop hypertension in the future. To date, non-drug treatment has been the main strategy for OHT in children.

## Author Contributions

JD and HJ contributed to the conception of the review, revision of the manuscript, and final approval of the version to be published. YH examined the literature and drafted the article. All authors contributed to the article and approved the submitted version.

## Conflict of Interest

The authors declare that the research was conducted in the absence of any commercial or financial relationships that could be construed as a potential conflict of interest.
